# Retinoic acid facilitates inactivated transmissible gastroenteritis virus induction of CD8^+^ T-cell migration to the porcine gut

**DOI:** 10.1038/srep24152

**Published:** 2016-04-15

**Authors:** Xiaojuan Chen, Chongzhi Tu, Tao Qin, Liqi Zhu, Yinyan Yin, Qian Yang

**Affiliations:** 1 Key Lab of Animal Physiology and Biochemistry, Ministry of Agriculture, College, China

## Abstract

The digestive tract is the entry site for transmissible gastroenteritis virus (TGEV). TGEV transmission can be prevented if local immunity is established with increased lymphocytes. The current parenteral mode of vaccination stimulates systemic immunity well, but it does not induce sufficient mucosal immunity. Retinoic acid (RA) plays an important role in the induction of cells that imprint gut-homing molecules. We examined whether RA assist parenteral vaccination of pigs could improve mucosal immunity. We demonstrated that elevated numbers of gut-homing CD8^+^ T cells (which express α4β7 and CCR9 molecules) were presented in porcine inguinal lymph nodes and were recruited to the small intestine by RA. Intestinal mucosal immunity (IgA titre) and systemic immunity (serum IgG titre) were enhanced by RA. Therefore, we hypothesized that RA could induce DCs to form an immature mucosal phenotype and could recruit them to the small intestinal submucosa. Porcine T-cells expressed β7 integrin and CCR9 receptors and migrated to CCL25 by a mechanism that was dependent of activation by RA-pretreated DCs, rather than direct activation by RA. Together, our results provide powerful evidence that RA can assist whole inactivated TGEV (WI-TGEV) via subcutaneous (s.c.) immunization to generate intestinal immunity, and offer new vaccination strategies against TGEV.

Transmissible gastroenteritis (TGE), which is caused by transmissible gastroenteritis virus (TGEV), is a highly contagious disease in newborn piglets^1^. After entering the digestive tract, TGEV can replicate in intestinal enterocytes and then induce enteritis and watery diarrhoea^2^. Both live and killed TGEV vaccines (intramuscular route or subcutaneous injection) are currently available to control TGE; however, they are not always successful^3^. These vaccination strategies can stimulate systemic immunity well; however, they do not induce sufficient mucosal immunity, especially the induction of local, virus-specific sIgA antibodies^4^. Determining how to induce a mucosal immune response and improve local immunity in the intestine is important in preventing enteropathogen infection.

Excellent induction of mucosal immunity depends on the inductive and effector sites^5^. The mucosal immune mechanism includes naive lymphocyte activation in classical inductive sites (such as intestinal Peyer’s patches), after which the sensitized lymphocytes migrate to the blood circulation before homing to effector sites (such as the intestinal epithelium or lamina propria) and differentiating into effector lymphocytes that contribute to immunity^6^. Effective viral clearance requires the rapid migration of effector T cells to the site of intestinal infection. Intestinal lymphocyte homing includes lymphocytes selectively passing through the postcapillary venule and migrating directly to the intestinal epithelium or lamina propria. T cells migrating to the intestine require the expression of specific receptors, including homing receptors, such as α4β7-integrin and CCR9, and their corresponding ligands (i.e., addressin-cell adhesion molecule 1, MAdCAM1) on endothelial cells from intestinal postcapillary venules^7^ as well as ligands (such as CCL25) on the intestinal epithelium^8^,^9^. CCR9/CCL25 interactions can induce the homing of effector T and B cells to the gut^10^,^11^. Additionally, these interactions can guide plasmacytoid dendritic cells (DCs) to the intestine^12^,^13^.

Retinoic acid (RA), a vitamin A metabolite, has emerged as a critical factor in mucosal immune responses^14^. RA induces intestinal cytokines generation^15^,^16^ and IgA responses^10^,^17^,^18^, and RA supplementation reduces morbidity and mortality due to enteric infectious diseases^19^. Furthermore, RA was shown to stimulate T cell proliferation^16^, up-regulate the expression of gut-homing receptors on lymphocytes, and promote their migration to the intestine^9^,^10^,^11^,^18^,^20^. Furthermore, mucosal DCs can augment the expression of integrin α4β7 and the chemoattractant receptor, CCR9 on activated lymphocytes in the presence of RA^18^, which mediates their homing to the gut mucosa^21^. Therefore, in our study, we used RA combined with whole inactivated TGEV (WI-TGEV) to immunize piglets via subcutaneous (s.c.) vaccination^22^ in order to induce T cell homing to the small bowel intestinal mucosa, as well as to generate more mucosal DCs. We found that these results will offer new approaches for the development of vaccine candidates against TGEV in newborn piglets.

## Materials and Methods

### Animals

Fifty TGEV-seronegative Yorkshire, Duroc, and Large White crossbred piglets at four weeks of age purchased from Huachen Pig Farm (Nanjing, China). The animal studies were approved by the Institutional Animal Care and Use Committee of Nanjing Agricultural University and followed the National Institutes of Health’s guidelines for the performance of animal experiments.

### Reagents

Retinoic acid (RA), 5-(and 6)-carboxyfluorescein diacetate succinimidyl ester(CFDA-SE), bovine serum albumin (BSA), LPS (from Escherichia coli 026:B6), were purchased from Sigma-Aldrich, Saint Louis, USA. FITC-conjugated mouse anti-pig CD8α (76-2-11) monoclonal antibody (mAbs), rat anti-mouse integrin β7 (NA/LE) mAbs, were purchased from BD Biosciences, USA. FITC-conjugated mouse anti-human CD16 (KD1) mAbs, FITC or PE-conjugated mouse anti-pig swine workshop cluster 3a (SWC3a) (74-22-15) mAbs, PE-conjugated mouse anti-pig CD1(76-7-4) mAbs, rabbit anti-human CCR9 (E99) mAbs, rabbit anti-human CCR9 mAbs (Extracellular domain), PE/Cy5-conjugated rat anti-mouse CD11b (M1/70) mAbs, rabbit anti-human CD3 (SP7) mAbs, RO 41-5253 were purchased from Abcam, Hongkong. FITC-conjugated mouse anti-pig SLA-DR (2E9/13) mAbs, PE-conjugated mouse anti-human HLA-DP (HL-38) mAbs were obtained from LifeSpan BioSciences, USA. Rabbit anti pig IgG, goat anti pig IgA antibody were purchased from Bethy laboratories, USA. PE-conjugated goat anti-rat IgG antibody was bought from Santa Cruz biotechnology, Texas, USA. Purified TGEV S-AD protein^23^. Purified porcine CCL25 protein was generated in our lab. DyLight 649-conjugated goat anti-rabbit IgG antibody, DyLight 488-conjugated goat anti-rabbit IgG antibody, DyLight 594-conjugated goat anti-rabbit IgG antibody were purchased from Multiscience, Hangzhou, China. ABC-based system (biotinylated goat anti rabbit IgG antibody) was used as the secondary antibody with DAB as a chromogen was (Boster, Wuhan, China). Porcine intestinal epithelial cell line (IPEC-J2) and swine testicle (ST) cell lines were purchased from Jennio Biotech, Guangzhou, China.

### Vaccine

The TGEV strain (SHXB, wild-virulent strain) was supplied by the Jiangsu Academy of Agricultural Sciences (Nanjing, China)^2^. The viral 50% tissue culture infectious dose (TCID_50_) of the SHXB TGEV strain was 1 × 10^7.4^ TCID_50_/100 ul. The viruses were inactivated by ultraviolet radiation (UV) for 4 h and tested for complete loss of infectivity by inoculation into ST cells for three passages^24^. RA was dissolved with dimethyl sulfoxide (DMSO) according to the manufacturer’s instructions. An RA working solution was diluted with corn oil (Changshouhua, Shandong, China).

### Immunization

Piglets were randomly divided into 5 groups, with 6 pigs per group. The first group was immunized via the subcutanoues route with 1 ml of corn oil into the right groin as a control, the second group was immunized via the s.c. route with 1 ml of RA (15 mg/ml in corn oil), the third group was immunized via the s.c. route with 1 ml of WI-TGEV (1 × 10^7.4^ TCID_50_/100 ul), the fourth group was immunized via the s.c. route with 1 ml of WI-TGEV (1 × 10^7.4^ TCID_50_/100 ul) combined with 1 ml of RA (15 mg/ml in corn oil), and the last group was orally immunized with 10 ml of WI-TGEV (1 × 10^7.4^ TCID_50_/100 ul) (Table 1). All of the piglets were immunized at the age of 35 days and given a booster immunization at the age of 42 days on the same side. All of the animals were anaesthetized and killed 28 days after the booster immunization.

### Sample collection

On days 7, 14, 21, 28, 35 after the first vaccination, four pigs were sampled randomly from each group for determination of serum and faecal TGEV-specific antibody. The serum was separated by centrifugation and stored at −70 °C for TGEV-specific IgG antibody detection. For TGEV-specific IgA antibody detection, 0.3 g faecal sample was resuspended with 1 ml of PBS, and the supernatant was collected by centrifugation and stored at −70 °C for TGEV-specific IgA antibody detection. On days 35 after first vaccination, pigs were exsanguinated, the ileum and the right side of the inguinal lymph nodes (ILNs) were stored in PBS. And then cells from the ileum and ILNs were isolated and analyzed by flow cytometry^25^. A portion of the ileum was triturated with a mortar and liquid nitrogen, and the homogenate was weighed and dissolved in PBS at a dilution of 1 mg per 5 μl. The supernatants of these homogenates were collected after centrifugation for the detection of specific IgA antibodies. After dilution, the homogenate supernatant protein concentrations were measured using a BCA protein assay kit (Thermo Scientific Pierce). The ileal tissue was either frozen in liquid nitrogen and stored at −70 °C for immunofluorescence detection or fixed in Bouin’s liquid for immunohistochemical detection.

### Flow cytometry analyses

Cells from the ileum and ILNs were isolated as previously described^26^. The cells were washed twice with cold PBS and stained with specific fluorescent antibodies at 4 °C for 0.5 h per the manufacturer’s guidelines. To determining the levels of homing receptor on CD8^+^ cells, cells were stained with antibodies against integrin β7(BD, 553812, 0.025 μg for 10^6^ cells), CCR9(Abcam, ab32556, 0.025 μg for 10^6^ cells) and CD8α (BD, 551303, 1 μg for 10^6^ cells). To examination of tissue DC phenotypes, cells were stained with antibodies against CD11b (Abcam, ab25533, 0.3 μg for 10^6^ cells) and SWC3a (Abcam, ab25684, 0.2 μg for 10^6^ cells), or CD16 (Abcam, ab124042, 10 μl for 10^6^ cells) and HLA-DP (LSBio, LS-C46457/49786, 20 μl for 10^6^ cells). To analysing BM-DC phenotypes, cells were stained with antibodies against CD1a and SWC3a (Abcam, ab24885, 1 μg for 10^6^ cells), HLA-DP and SWC3a, or integrin β7, CCR9 and SWC3a. The respective isotype controls were used as negative controls. After three PBS washes, in each sample were acquired 1 × 10^4^ cells by flow cytometry with a BD FACS Calibur (BD Biosciences, US)^25^,^27^. Data were analyzed using FlowJo 7.6 (Tree star).

### SIgA and CD3^+^ T lymphocyte detection

Paraffin-embedded ileal sections were dewaxed in xylene and rehydrated in decreasing concentrations of ethanol^2^. After being blocked with 5% bovine serum for 30 min, the sections were incubated with the primary antibodies overnight at 4 °C, followed by incubation with secondary antibodies at room temperature for 1 h. The IgA^+^ cells were labelled with goat anti-pig IgA followed by Alexa Fluor 488 donkey anti-goat IgG. The CD3^+^ cells were labelled with rabbit anti-human CD3 IgG followed by biotinylated goat anti-rabbit IgG, and then the sections were sealed with a coverslip for examination. The respective isotype controls were used as negative controls. The sections were visualized with an Axioplan 2 microscope (Zeiss, Oberkochen, Germany), 400× magnification.

### Specific IgG and IgA detection

The TGEV-specific IgG and TGEV-specific secretory IgA (sIgA) levels were measured with enzyme-linked immunosorbent assays (ELISAs) as previously described^28^. Briefly, ELISA plates were coated with 0.3 μg of purified recombinant TGEV S-AD protein (the major antigenic sites A and D in TGEV)/well at 4 °C overnight. Following protein removal, the plates were blocked with 0.6% (wt/vol) bovine serum albumin (BSA) in PBS for 2 h at 37 °C and then incubated with 100 μl of samples for 1 h at 37 °C. After washing with PBST, 100 μl of HRP-conjugated rabbit anti-pig IgG or goat anti-pig IgA antibody was added at a 1:10,000 dilution and incubated for 1 h at 37 °C. The plates were washed 5 times and incubated with 3,3′,5,5′-tetramethylbenzidine (TMB). After 10 minutes, the reaction was arrested with sulphuric acid (2 M), and the absorbance was read at 495 nm with a microplate reader.

### Isolation and culture of bone marrow-derived dendritic cells (BM-DCs)

Swine BM-DCs were isolated as per our advanced methods^25^. Briefly, bone marrow was extracted from the femurs of piglets and treated with red blood cell lysing buffer. The bone marrow cells were differentiated into DCs by resuspending the cells in complete medium (RPMI-1640 (Invitrogen) supplemented with 10% foetal bovine serum (FBS) (Wisent, CA), 1% penicillin/streptomycin, 10 ng ml^−1^ porcine granulocyte-macrophage colony-stimulating factor (GM-CSF), and 10 ng ml^−1^ porcine IL-4 (Prospec, Ness Ziona, Israel) and plated at 1 × 10^6^ cells per ml in 6-well plates. Non-adherent granulocytes were removed by discarding the culture medium after 60 h of culture. On day 6 of culture, the clusters were harvested and subcultured overnight so that the adherent cells could be removed. Non-adherent cells were collected after 7 days of culture, washed, and used as immature DCs for subsequent studies.

### *In vivo* homing assay

Three piglets were used *in viv*o homing assay. Porcine peripheral blood mononuclear cells (PBMCs) were isolated from the blood of piglets by density centrifugation using Histopaque (1.077 g l^−1^) (Sigma). To isolate T cells, PBMCs were labelled with a mouse anti-CD3 antibody (Abcam, Hong Kong) followed by incubation with rat anti-mouse IgG1 microbeads (MACS; Miltenyi Biotec, Germany)^2^. CD3^+^ T cells were cultured with or without RA for 48 h. Thereafter, the cells were divided into two parts, one part was detected with flow cytometry to determining the homing receptor levels on CD8^+^ cells, cells were stained with antibodies against integrin β7, CCR9 and CD8α, and the other part was harvested for the *in viv*o homing assay. For the *in viv*o homing assay, the cells treated with RA were labelled with Cell Tracker CM-DiI (Life Technologies, Eugene), and the cells treated without RA were labelled with CFDA-SE (Invitrogen), respectively, according to the manufacturer’s instructions. Thereafter, the cells were centrifuged and extensively washed. Then, 120 million cells from each preparation were mixed and intravenously injected into recipient piglets (n = 3). The recipients were sacrificed 24 h after the injection. The number of input cells was counted with flow cytometry. For the flow cytometry analyses, cells were isolated from the ileum. For the histological analyses, ileum blocks containing Peyer’s patches were immersion fixed in 4% paraformaldehyde. The fixed tissues were washed with 0.1 M PBS (pH 7.4), embedded in O.C.T. compound (Sakura Finetechnical, Tokyo, Japan), and finally stored at −70 °C. The ileal tissues were cut into 10 μm sections, frozen and mounted on poly-L-lysine-coated glass slides. The Tracker CM-DiI- or CFSE-labelled cells were observed with an Axioplan 2 microscope (Carl Zeiss, Oberkochen, Germany).

### Mixed lymphocyte reaction (MLR)

CD3^+^ T cells (isolated from PMBCs with the same treatment as above) were labelled with 0.1 μM CFDA-SE. Purified BM-DCs were pulsed with varying concentrations of RA or 100 ng/ml LPS for 48 h before use. Following repeated washes, T cells, as responder cells, were incubated with pre-treated BM-DCs at 1 × 10^6^/well (DC/T-cell ratios of 1:1 and 1:10) for 5 days. Thereafter, the mixed cells were detected with flow cytometry for T-cell proliferation and the expression of gut-homing receptors on T cells.

### Construction of porcine CCL25

The DNA fragment of porcine CCL25 (NM_001025214.1, Life Technologies, Eugene) was amplified from porcine genome used the primers F-GGACTCAGATCTCGAGGCCACCATGAGGCCGTGGCTCCTGGCC and R-TCTGGAACATCGTATGGGTATGGTCCTGGAATAGCTGTTG, and inserted into the lentiviral vector pLVX-AcGFP1. Lentiviral production was achieved through calcium phosphate transfection of pLP1, pLP2, pLP/VSVG (Invitrogen) plasmids. Lentivirus packaging referenced literature^29^. For infection 1 × 10^5^ IPEC-J2 cells were plated per 9 cm^2^ dish a day prior to infection. At the day of infection the viral supernatant was supplemented with Polybrene (8 mg/mL, Life Technologies, Eugene) and added to the cells for 8 h. The multiplicity of infection was set to 1 to obtain single copy integration of the synthetic gene circuit. Porcine CCL25 high-expressed clone was cultured to collect supernatant and activity was assayed by NanoDrop 2000 UV-Vis Spectrophotometer (Thermo Scientific, USA).

### Transwell migration assay

Migration assays were performed as previously described^8^ using 24-well Falcon cell culture inserts with 8 μm pores (Corning, New York, USA). CD3^+^ T cells were isolated from PMBCs with the same treatment as above. RA, RO 41-5253 or RA plus RO 41-5253 were added to the BM-DCs at different concentrations for 48 h before use. Following repeated washes, pre-treated BM-DCs were mixed together with T cells at 1 × 10^6^/well (DC/T-cell ratios of 1:1) for 5 days. Thereafter, the mixed cells were collected for transwell migration assays. To induce cell migration RPMI-1640 medium (900 μl) containing CCL25 (10 ng/ml) was placed in the lower chamber, and RPMI-1640 medium (350 μl) containing 1 × 10^6^ mixed cells (DCs mixed with T cells) was placed in the upper chamber. After 4 h of incubation at 37 °C, to determine the amount of CD3^+^ cells in the lower chamber, the migrated cells were collected, we counted the number of CD3^+^ cells by flow cytometry.

### Statistical analysis

All of the data were expressed as the means ± S.D. and analyzed with SPSS 17.0. The data were analyzed with non-parametric tests, the unpaired Mann-Whitney for the *in vivo* data and the paired Wilcoxon for the comparisons between conditions with cells from the same piglet. A *P* value < 0.05 was considered statistically significant.

## Results

### RA increases the frequency of gut-homing cells in piglets immunized with WI-TGEV

**Figure 1:**
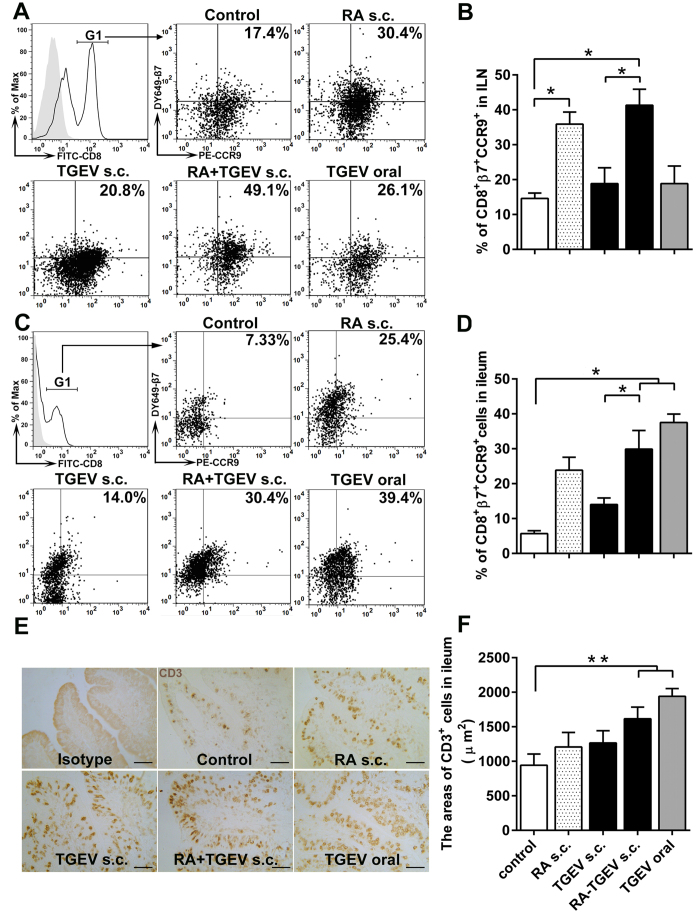
Gut-homing-specific molecules are increased on CD8^+^ T cells after subcutaneous immunization with RA plus whole inactivated TGEV virus (WI-TGEV) in piglets. (**A**–**D**) Cells were isolated from the porcine ileum and inguinal lymph nodes (iLNs) and analyzed by flow cytometry. The cells were gated based on the CD8^+^ T cells (G1), and the gated cells were further selected based on their β7-integrin^+^ and CCR9^+^ expression^27^. (**A**,**B**) iLNs. (**C**,**D**) ileum. (**D**,**F**) Ileal sections were immunohistochemically stained for CD3^+^ T cells. (**F**) The results are expressed as the area of positive cells in a random field *P < 0.05; **P < 0.01. The data represent the means ± S.D. of four samples. Scale bars: 50 μm.

In the intestinal mucosal immune system, cellular immune responses play an important central role in the outcome of several viral infections^30^. Virus-specific CD8^+^ T cells play a central role in controlling and eliminating most pathogen infections. To the best of our knowledge, there are limited reports of studies focused on the design of CD8^+^ T cells-based vaccines in mucosal immunity^31^,^20^. When given together with RA, exogenous antigens can effectively stimulate lymphocytes, which have an increased expression of gut-homing receptors and decrease the expression of skin-homing receptors on lymphocyte surfaces. These research studies indicated that the skin could replace Peyer’s patches as an outstanding mucosal immune inductive site^32^. As we known, the area of abdominal skin was large and the temperature was constant, which was conducive to the vaccine absorption. Furthermore, there was less nerves distribution in abdominal skin, which was beneficial to reduce the injection pain and stress. Inguinal lymph node was located in the groin, so in our study the skin on the right groin was selected as an immune inductive site. We subcutaneously immunized piglets with corn oil, RA, or WI-TGEV alone or in combination with RA, or we orally immunized piglets with WI-TGEV alone. To analyze whether RA has the capability of generating gut-homing cells; therefore, cells were isolated from the right side of the inguinal lymph nodes (ILNs) and ileum after immunization. Intriguingly, the flow cytometry data from the ILNs showed that the number of CD8^+^ cells that expressed β7-integrin and CCR9 was significantly increased after RA administration compared with the control (Fig. 1A,B, *P* < 0.05). Furthermore, the s.c. administration of RA plus TGEV increased the number of gut-homing CD8^+^ cells expressing β7-integrin and CCR9 compared with the s.c. administration of TGEV alone in the ILNs (Fig. 1A,B, *P* < 0.05).

Lymphocyte homing to the small intestine is believed to play a crucial role at mucosal immunity effector sites, and CD8^+^ effector T cells in intestinal epithelium can effectively prevent or directly limit infection at the entrance site of enteric viruses^33^. Furthermore, effector T cells maintain the epithelial barrier and tissue homeostasis^34^. In our study, the ileum was selected as the mucosal immune effector site. Additionally, we assessed the level of CD8^+^ cell homing to the ileum. Notably, the flow cytometry data from the ileum showed that following the RA plus TGEV s.c. and oral TGEV treatments, the gut tropism receptor frequency on CD8^+^ cells was remarkably enhanced compared with the controls (Fig. 1C,D, *P* < 0.05). Next, we further evaluated the number of CD3^+^ cells in the porcine ileum with immunohistochemistry. In the cross-sectional view, CD3^+^ T cells were gathered in the epithelial layer and villous lamina propria (Fig. 1E, 400× magnification). The RA plus TGEV s.c. administration substantially expanded the areas with CD3^+^ T cells compared with the controls (Fig. 1F, *P* < 0.01), although these levels were lower than those observed with the oral TGEV treatment.

### RA assists WI-TGEV in enhancing both local and systemic immune responses after subcutaneous immunization in piglets

**Figure 2:**
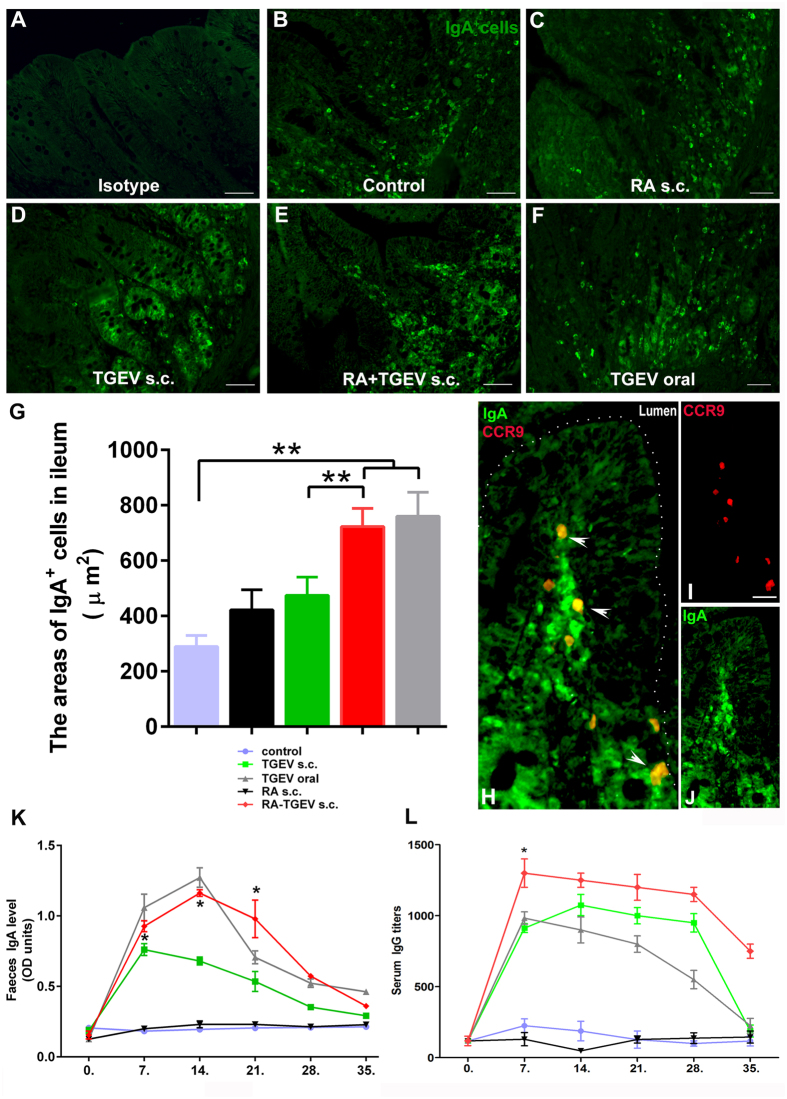
RA assists TGEV in enhancing both local and systemic immune responses after subcutaneous immunization in piglets. (**A**–**F**) Ileal sections were immunofluorescently stained for IgA^+^ cells (Alexa Fluor 488, green), which were mainly present in the lamina propria of the ileum. (**A**) Isotype. (**B**) Control. (**C**) RA s.c. (**D**) TGEV s.c. (**E**) RA plus TGEV s.c. (**F**) TGEV oral. (**G**) Quantification of IgA^+^ cell areas in the ileum. (**H**–**J**) Ileal sections from the s.c. RA plus TGEV group were stained for IgA^+^ (Alexa Fluor 488 green) and CCR9^+^ (DyLight 594 red) cells. (**I**) CCR9 channel. (**J**) IgA channel. (**L**) ELISA detection of antigen-specific IgA titres in faeces (n = 4). (L) ELISA detection of antigen-specific serum IgG titres (n = 4). The data represent the means ± S.D. of four samples. *P < 0.05; **P < 0.01. Scale bars: 50 μm.

Next, to verify whether RA could assist WI-TGEV in enhancing the mucosal and systemic immune responses, we determined the local secretion IgA and systemic IgG, immunization methods as above. Our immunofluorescence results showed that the IgA-secreting cells were mainly gathered in the villous lamina propria or around the ileal glands (Fig. 2B–F). In the ileum, the areas of IgA-secreting cells of the RA plus TGEV s.c. and oral TGEV treatment were significantly increased than control (*P* < 0.01), and the RA plus TGEV s.c. treatment significantly increased the areas containing IgA-secreting cells compared with the TGEV s.c. alone treatment (Fig. 2G, *P* < 0.01), however, this level was slightly less than the level observed following the oral TGEV delivery. Furthermore, we noted that the IgA-secreting cells displayed IgA and CCR9 co-expression in the villous lamina propria after the RA plus TGEV challenge (Fig. 2H–J). A similar finding was observed in the faeces, in which the subcutaneous RA plus TGEV immunization obviously increased the TGEV-specific IgA levels compared with the s.c. TGEV alone treatment at days 7, 14 and 21(ELISA method) (Fig. 2K, *P* < 0.05), and this effect lasted longer than that of the oral TGEV immunization. For the systemic immune response, in the serum, we found that the TGEV-specific IgG titres in the RA plus TGEV s.c. treatment group remained at a high level during the entire immunity period (7–35 days) and were substantially greater than those in the other groups at day 7 (Fig. 2L, *P* < 0.05). These observations demonstrated that RA-assisted TGEV immunization could recruit IgA-secreting cells to the ileum and induce a robust antigen-specific IgA response in the faeces.

### RA assists TGEV s.c. treatment by increasing the DC numbers in the intestinal lamina propria of piglets *in vivo*

**Figure 3:**
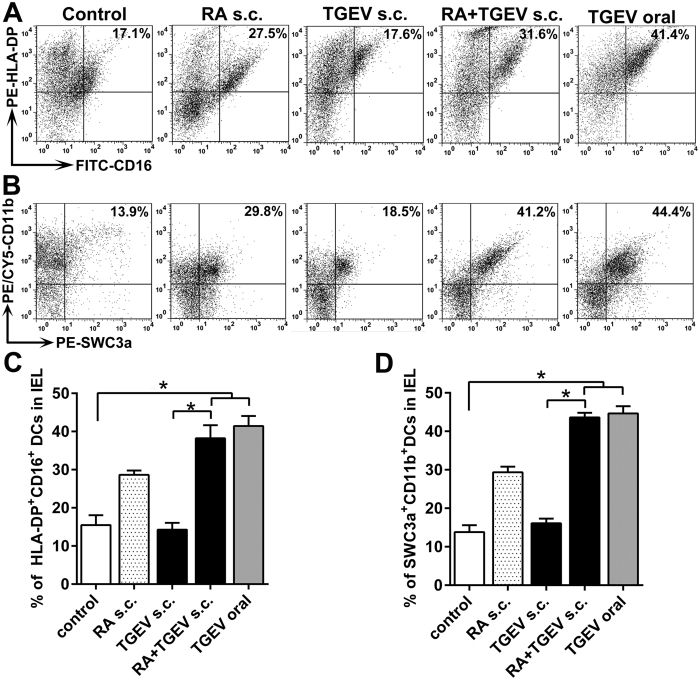
RA assists TGEV in enhancing the percentages of HLA-DP^+^ CD16^+^ and CD11b^+^ SWC3a^+^ intestinal DCs *in vivo*. On day 35 after the primary immunization, the percentages of PE-HLA-DP^+^ FITC-CD16^+^ (**A**,**C**) and PE/Cy5-CD11b^+^ PE-SWC3a^+^ (**B**,**D**) DCs in intestines from the immunized piglets were analyzed with flow cytometry. *P < 0.05. The data represent the means ± S.D. of three samples.

DCs are the main cellular element that controls T lymphocyte activation and regulation^35^, which is critical for subsequent immunity responses of the intestinal mucosa. Porcine small intestine DCs are express MHCII (SLA-DP), CD172a (SWC3a), CD16, CD11R1, or CD11b^2^,^26^. To determine whether RA in the vaccine could recruit DCs to the small intestine, we isolated intestinal cells from piglets and determined the DC numbers in the lamina propria by flow cytometry. Very few HLA-DP^+^ CD16^+^ DCs (Fig. 3A,C) or SWC3a^+^ CD11b^+^ DCs (Fig. 3B,D) migrated to the small intestinal lamina propria following TGEV s.c. treatment. Nevertheless, the s.c. administration of RA plus TGEV and oral TGEV (positive control) significantly enhanced the frequency of these migrated DCs compared with TGEV s.c. treatment (Fig. 3C,D, *P* < 0.05). These findings suggested that s.c. RA treatment could effectively facilitate TGEV not only by increasing the CD8^+^ cell numbers (Fig. 1C,D) but also by increasing the DC numbers in the lamina propria of the ileum.

### RA does not directly increase expression of gut-homing receptors on BM-DCs

**Figure 4:**
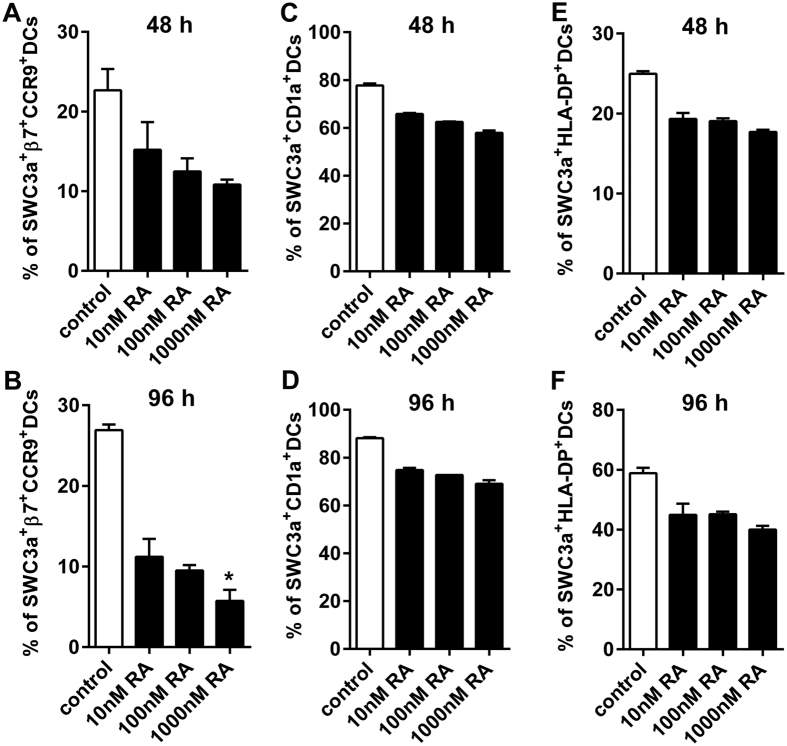
Induction of an immature BM-DC phenotype by RA *in vitro*. BM-DCs were treated with or without RA (10–1,000 nM). After 48 or 96 h of treatment, the cells were collected and measured with flow cytometry. (**A**) Quantification of the percentages of PE-β7-integrin^+^ and DyLight 649-CCR9^+^ cells among the FITC-SWC3a + BM-DCs after 48 h. (**B**) Quantification of the percentages of PE-β7-integrin^+^ and DyLight 649-CCR9 + cells among the FITC-SWC3a + BM-DCs after 96 h. (**C**) Quantification of the percentages of FITC-SWC3a + PE-CD1a + BM-DCs after 48 h. (**D**) Quantification of the percentages of FITC-SWC3a + PE-CD1a + BM-DCs after 96 h. (**E**) Quantification of the percentages of FITC-SWC3a + PE-HLA-DP + BM-DCs after 48 h. (**F**) Quantification of the percentages of FITC-SWC3a + PE-HLA-DP + BM-DCs after 96 h. *P < 0.05. The data represent the means ± S.D. of three samples. The results shown are from a representative experiment out of three experiments.

*In vivo*, we observed that RA-assisted TGEV treatment increased the DC numbers in the porcine intestine; however, the mechanism impacts DCs migration is unclear. A previous study showed that RA induced gut-homing receptors on mice BM-DCs in a narrow time window and had a stringent dose response^8^. Thus, we examined whether RA could induce porcine BM-DCs to express gut-homing receptors. After we directly treated porcine BM-DCs with RA for 48 or 96 h *in vitro,* the expression levels of the gut-homing receptors integrin β7 and CCR9 were decreased in a dose-independent manner (Fig. 4A,B), 1000 nM RA treatment significantly reduced the expression of the gut-homing receptors on DCs compared with controls (*P* < 0.05).

Next, to test whether RA could affect porcine BM-DC maturation, we analyzed the expression of the phenotype markers CD1a and HLA-DR. After BM-DCs were incubated with RA for 48 or 96 h, the percentages of SWC3a^+^ CD1a^+^ (Fig. 4C,D) and SWC3a^+^ HLA-DR^+^ (Fig. 4E,F) DCs were decreased in a dose-independent manner, but there was no significant difference.

### RA does not directly increase expression of gut-homing receptors on unactivated T cells

**Figure 5:**
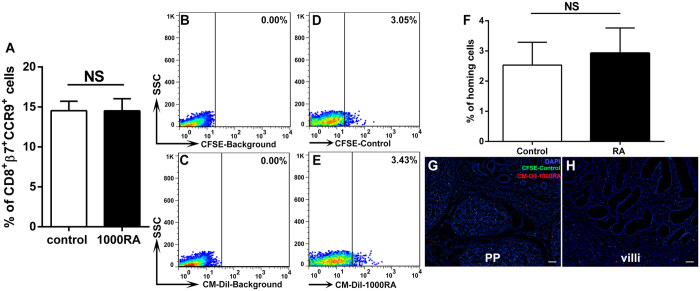
Direct interactions between RA and unactivated T cells with regard to their gut-homing properties. CD3^+^ T cells were treated with or without RA (1,000 nM) for 2 days. (**A**) Gut-homing specificity was examined by measuring the expression of PE-β7-integrin^+^ DyLight 649-CCR9^+^ on FITC-CD8^+^ T cells with flow cytometry. (**B**–**H**) In the recipient assay, RA-untreated T cells were labelled with CFSE (green), and RA-treated T cells were labelled with tracker CM-DiI (red). Equal cell numbers (1 × 10^8^) from each treatment were mixed and injected into donor pigs. (**B**–**E**) The cells that homed to the ileal villi were analyzed by flow cytometry; (**B**) CFSE-background. (**C**) CM-DiI-background. (**D**) The percentage of control (RA-untreated) cells (CFSE) in the ileum (**E**). The percentage of RA-treated cells (CM-DiI) in the ileum (**F**). The numbers of homing cells were quantified. Frozen ileal sections were stained with DAPI (blue) and observed with fluorescent microscopy. (**G**) PPs. (**H**) villi. The data represent the means ± S.D. of three samples. NS = no significance. Scale bars: 50 μm.

To better understand whether RA could directly imprint gut-homing-specific receptors on porcine T cells, unactivated T cells were directly treated with or without RA *in vitro*, and then the homing receptors β7 integrin and CCR9 were detected with flow cytometry. However, there was no significant difference between their levels of homing receptor levels on CD8^+^ cells with (up to 1000 nM) or without RA treatment (Fig. 5A).

Next, to evaluate whether RA-treated T cells could preferentially home to the small intestine, we performed competitive homing assay *in viv*o. T cells were isolated from a piglet and treated with or without RA, labelled with CM-DiI (red, molecular probes) or CFSE (green) for long-term cellular labeling. Equal numbers of cells from the two populations were mixed and adoptively transferred into piglets via intravenous injection. Twenty-four hours later, the mixed cells that homed to the ileal villi were detected by flow cytometry. However, the two populations homed equally into the ileum. There was no significant difference between the levels of homing cells in the piglets with or without RA-treated T cells (Fig. 5B–F). Observations of the intestinal villi and Peyer’s patches (PPs) in cryosections further confirmed the flow cytometry results of homing cells (Fig. 5G,H). These data of T-cell gut-homing further confirmed the above results that RA did not directly increase expression of gut-homing receptors on T cells. For a deficiency of gut-homing receptors on T cells, T cells could not recruited into ileum. Thus, RA did not directly induce T-cell homing to the gut in the piglets.

### RA-pretreated BM-DCs are required for inducing unactivated T cells to express high gut-homing-specific receptor levels

**Figure 6:**
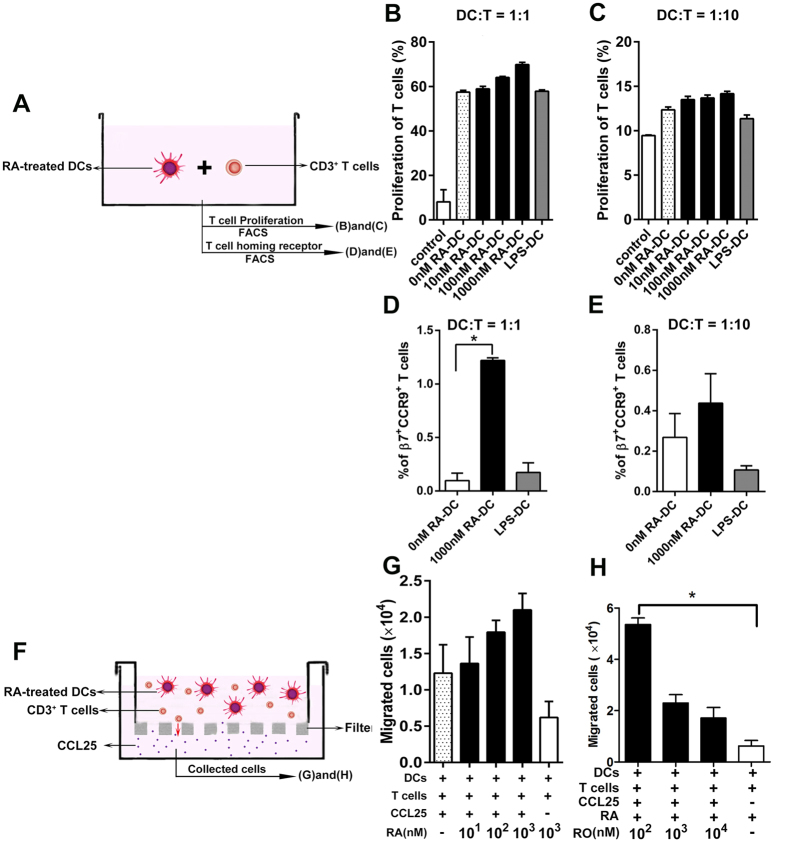
The role of DCs in T-cell proliferation, expression of gut-homing-specific molecules, and migration ability after RA treatment. (**A**) The experimental setting for the study of CD3^+^ T cells that were activated and expanded with BM-DCs. BM-DCs were treated with or without RA, or with LPS (100 ng/ml) for 2 days and then co-cultured with CD3^+^ T cells for 5 days. (**B**,**C**) In MLR experiments, RA-treated BM-DCs were used in two graded cell numbers to stimulate CFSE-labelled CD3^+^ T cells (DC:T cell ratios, 1:1 (**B**) or 1:10 (**C**)), and T-cell proliferation was detected with flow cytometry. (**D**,**E**) The expression levels of gut-homing-specific PE-β7-integrin^+^ DyLight 649-CCR9^+^ on FITC-CD8^+^ T cells were analyzed with flow cytometry. DC:T-cell ratios, 1:1 (**D**) or 1:10 (**E**). (**F**) The schematic depicts co-cultured cells (RA-treated BM-DCs and CD3^+^ T cells 1 × 10^6^) that were resuspended in 0.35 ml of RPMI 1640 medium and loaded onto the apical side of transwells (Corning, Lowell, MA; pore size, 8 μm). Then, 0.9 ml of medium or medium plus CCL25 (10 μg/ml) was added to the transwells. The numbers of cells that migrated to the basolateral side were measured. (**G**) Co-culture of CD3^+^ T cells and RA-treated BM-DCs, (**H**) co-culture of CD3^+^ T cells and RA and/or RO 41-5253-treated BM-DCs. *P < 0.05. The data represent the means ± S.D. of three samples. The results shown are from a representative experiment out of three.

In an MLR assay, RA-treated DCs cocultured with CD3 T cells, flow cytometry analyses were performed by collecting the mixed cells after 5 days incubation to determine T-cell proliferation and the homing receptor expression levels on T cells (Fig. 6A). The results showed that the BM-DCs treated by RA stimulated T-cell proliferation were increased in a dose-independent manner at BM-DC/T-cell ratios of 1:1(Fig. 6B) and 1:10 (Fig. 6C), but there was no significant difference compared with the untreated BM-DC group. Additionally, the β7 integrin and CCR9 expression levels on T cells were detected with flow cytometry. The data showed that 1,000 nM RA-treated BM-DCs induced CD3^+^ cells to highly express β7 integrin and CCR9 compared with the untreated BM-DC group (Fig. 6D, *P* < 0.05, BM-DC/T-cell ratios of 1:1). At BM-DC/T-cell ratios of 1:10, 1,000 nM RA-treated BM-DCs still induced CD3^+^ cells to highly express β7 integrin and CCR9, but there was no significant difference compared with the untreated BM-DC group (Fig. 6E).

To verify whether the porcine cells that expressed CCR9 could respond to CCL25 (CCR9 ligand), we evaluated the migration capacity of T cells towards CCL25 (Fig. 6F), which is constitutively expressed by intestinal epithelial cells^9^. All of the T cells when activated by RA-activated DCs showed strong responsiveness to CCL25, the number of migrated cells with an RA dose-independent increase (Fig. 6G). Notably, the maximum responsiveness to CCL25 relied on exogenous RA (1,000 nM) conditioning. On the contrary, the ability of the T cells to migrate from the apical side to the basolateral side of the transwell filters was significantly reduced after RA receptor agonists (RARs) (Ro-41-5253, 1,00 to 10,000 nM) were added with RA (1,000 nM) to the culture (Fig. 6H), the number of migrated cells among 1,000 or 10,000 nM Ro-41-5253 plus RA or RA treatments was no significant difference, which was consistent with a previous observation^18^. Taken together, the cell migration assay results revealed a strong chemotactic response in T cells when activated by RA-activated DCs. These data demonstrated that RA-pretreated BMDCs could activate T cells to express high functional levels of the gut-homing receptor CCR9, as well as to migrate towards the porcine chemokine CCL25.

## Discussion

Current TGEV vaccines are effective in protecting neonatal piglets from TGEV infection. Two types of TGE vaccine are currently licensed for commercial use, including live attenuated vaccines, which are delivered orally, and inactivated vaccines, which are administered via parenteral inoculation to induce immunity^3^. Although live attenuated vaccines induce efficient protective immunity, live viruses are unsafe and can revert to virulence. In the development of a vaccine, safety and effectiveness are important considerations. In recent years, inactivated vaccines have been known for their safety and have been given higher priority in the pig industry, but inactivated vaccines require multiple booster immunizations and/or supplementation with adjuvants, because they are less immunogenic than live vaccines.

The initial TGEV infection occurs in the digestive tract. The oral route of vaccine delivery should be an effective method to prevent viral adhesion and colonization, thus decreasing viral shedding in the digestive tract^28^. However, the oral route for vaccine delivery is the most challenging and difficult to achieve, particularly for inactivated vaccines. The efficacies of oral inactivated vaccines are currently poor, mainly because inactivated antigens must overcome a series of mucosal barriers, including mucus, digestive fluid, and compact epithelium, before they are captured by submucosal antigen-presenting cells (APCs)^27^. Thus, oral administration generally requires a large concentration of antigens to achieve effective immune protection levels^36^. In this study, the antigen dose for the oral immunization (positive control) was 10 times greater than that of the subcutaneous immunization, which significantly increased the production cost of the vaccines. An ideal TGEV vaccine should induce both local mucosal and systemic immune responses.

One effective strategy to improve local immunity involves inducing lymphocyte trafficking to the intestinal mucosa. RA is critical for inducing gut-homing receptors on T cells, which enhances their migration capacity to the intestinal mucosa^11^. Our data confirmed that RA plus TGEV s.c. could generate a great number of gut-homing CD8^+^ T cells in the inguinal lymph nodes below the subcutaneous immunization site. This was consistent with a previous report that subcutaneous RA-assisted antigen treated mice had increased lymphocytes at the injection site lymph nodes and these cells had up-regulated gut-homing receptor levels and down-regulated skin-homing receptor levels^32^. These results suggested that RA played an important role in immune induction.

CD8^+^ effector T cells in the intestinal epithelium can effectively prevent or directly limit enteric virus infection^33^, as well as maintain the epithelial barrier and tissue homeostasis^34^. The *in vivo* assay in our study demonstrated that using RA plus TGEV s.c. could greatly increase the number of CD8^+^ cells expressing β7 integrin and CCR9 in the ileum. However, the CD8^+^ cells in the s.c. TGEV alone treated mice did not show significant changes, indicating that the RA was necessary to see CD8^+^ cell increase in β7 integrin and CCR9. Furthermore, it was recently reported that CCR9 expression on T cells may also regulate the localization and ability of mature CD8αα intestinal intraepithelial lymphocytes in the small intestinal epithelium^37^ to influence mucosal immune responses. CD8αα T cells of intraepithelial lymphocytes (IEL) that likely function as regulatory T cells^38^, these CD8αα T cell subsets in gut maintain gastrointestinal homeostasis^39^. In agreement with the migration of gut-homing CD8^+^ cells, our observations showed that after RA plus TGEV s.c. treatment, many CD3^+^ T cells were recruited to the intestinal villi and lamina propria, and these data also showed that RA-assisted antigen s.c. can establish a stronger and faster cellular immune response to defend against foreign pathogens^40^.

SIgA plays an important role in reducing viral adhesion and capturing invasive viruses^28^. Thus, a large amount of IgA-secreting cell homing to the gut is important to produce IgA antibody against intestinal pathogens^41^. RA is required to induce gut-tropic IgA-secreting cells in mice and humans^32^,^42^,^43^. In our study, IgA-secreting cells were, remarkably, recruited to the intestinal lamina propria after the s.c. RA-TGEV treatment, which was in line with the subsequent strongly initiated IgA antibody response in the local porcine intestinal mucosa. Furthermore, the CCR9 expression on the IgA-secreting cells helped their cellular recruitment, and this was consistent with a previous report that RA was critical for the generation of gut-homing receptors on IgA-secreting cells^41^. Interestingly, RA plus TGEV immunization generated a long-lasting specific-sIgA response in the faeces, even though it was lower than the levels following the oral immunization, implying that an enhancement in SIgA secretion may be conducive to TGEV eliminate and infection resistance in the gut. However, it is worth mentioning that the antigen dose in the oral immunization was 10 times higher than that in the subcutaneous immunization in our study. Thus, at equal amount of antigen, it is possible that RA addition increases IgA secretion even above the secretion seen in response to the oral vaccine. Therefore, the RA administration in the subcutaneous immunization not only improved TGEV mucosal humoral immune responses but also was less expensive. Moreover, serum IgG plays a vital role in systemic immune responses, particularly for protecting against invasive pathogens that enter the bloodstream^44^. RA combined with the s.c. TGEV injection indeed induced a pronounced IgG response, which could compensate for the disadvantages of the oral immunization.

DCs have unique capacities to induce primary T-cell responses^35^,^45^,^46^. RA has an important influence on DC differentiation, maturation and functions, such as the capacity for DCs to induce IgA in Peyer’s patches^47^. In our study, we found that RA could induce WI-TGEV to increase the DC number in the intestinal lamina propria of piglets *in vivo*. The immature DCs (iDCs) that are located beneath the intestinal epithelium play an important role in monitoring^48^ and capturing antigen^49^. This implied that the DCs that were recruited following the RA-assisted TGEV s.c. treatment had the characteristics of immature DCs, as they patrolled the intestinal tract, captured antigens in the intestinal lumina, quickly migrated to MLNs for antigen presentation^25^ and improved the porcine mucosal immunity.

DCs are derived from proliferating precursors in the bone marrow that migrate via the blood to lymphoid and non-lymphoid tissues^50^. To investigate the mechanisms by which RA acted on DCs, we directly treated porcine BM-DCs with RA (*in vitro*), However, the RA treatment was unable to up-regulate the gut-homing-specific molecules as in mice^8^, even with a wide range of doses. The possible reason for this may have been due to the species differences between porcine and mice. Our results also showed that RA couldn’t up-regulated the expression of maturation phenotype molecules on porcine BM-DCs *in vitro*, such as SWC3a, CD1a, and HLA-DR molecule expression; these findings were supported by a previous study showing that RA treatment endowed BM-DCs with immature phenotypes via the down-regulation of CD80/86, CD40, and MHCII expression in mice^51^. These observations suggest that RA inhibits porcine BM-DC maturation, and they may help explain our previous observation that the DCs recruited to the intestinal lamina propria following RA-TGEV treatment were immature DCs that were conductive to capturing antigen and initiating a specific antibody response.

Given that RA failed to directly induce gut-homing receptors on DCs, we wondered whether RA could induce porcine lymphocytes to express the integrin β7 and CCR9 receptors. However, in the porcine *in vitro* and competitive homing experiments, RA was unable to directly induce unactivated T cells to express gut-homing-specific receptors, and it also failed in inducing T-cell migration to the small bowel. We wondered about the mechanisms that induce CD8^+^ T cells to express the integrin β7 and CCR9 receptors after RA-assisted TGEV s.c. challenge *in vivo*. Some studies have suggested that DCs are crucial in regulating downstream activation and differentiation of T cells^52^,^53^. Moreover, some evidence has demonstrated that CD8^+^ T cells and IgA-secreting B cells express gut-homing receptors via intestinal DC interactions^10^,^54^. The above analysis tempts us to speculate that RA may regulate DCs in the porcine inguinal lymph nodes to imprint gut tropism receptors on lymphocytes. Intriguingly, our data confirmed that RA cooperated with BM-DCs in promoting T cell proliferation, suggesting that RA-pretreated BM-DCs may have a powerful capacity to transfer antigens to T cells. Our data further indicated that T cells expressed high gut-homing-specific receptor levels following incubation with RA-pretreated BM-DCs, implying that porcine RA-pretreated BM-DCs may have the ability to imprint integrin β7 and CCR9 receptor expression on lymphocytes. Moreover, a cell migration assay was performed using transwell inserts, and the T cells that were treated with RA and DCs displayed a strong chemotactic response to porcine CCL25, which was consistent with a recent study in mice^8^ that showed a powerful homing capacity for these cells. Additionally, the capacity of RA-pretreated DCs to induce gut-homing receptors on responding T cells was blocked by an RAR antagonist, and those T cells lost the ability to migrate, supporting the hypothesis that RA is indispensable for generating gut tropic T cells in piglets. In view of the *in vitro* study, the RA-pretreated BM-DCs showed a significant ability to activate T cells to express gut-homing receptors for migration. One possible mechanism for this, with regard to the RA-pretreated DCs, is that RA could act as a “mucosal adjuvant” to induce mucosal DC and lymphocyte activation^18^,^54^. Furthermore, exogenous RA promotes intestinal lamina propria DCs to produce endogenous RA^55^, which guarantees gut-homing α4β7 and CCR9 expression by effector T cells^11^. Thus, another possibility is that the RA-pretreated DCs may also generate or combine with other cytokines to jointly promote gut-homing molecule expression, such as RA inducing mucosal DCs to secrete TGF β and IL-6, which enhance gut-homing receptors on lymphocytes^18^. However, the mechanistic knowledge by which porcine intestinal DC subsets develop is still limited; therefore, further studies will be needed to obtain insights into these potential mechanisms.

Viral diarrhoea is the second major cause of death and a main reason for malnutrition in children under five years of age. Compared to murine models, porcine models have substantial advantages, including physiological, anatomical and genetic similarities to the human intestine as well as defence mechanisms against disease similar to those of humans^56^,^57^, and these models have recently been widely applied to studies of intestinal immunity^58^, gut microbiota^59^, nosogenesis of rotavirus infection in newborns^60^, and even organ transplantation^61^. Our research regarding s.c. RA-assisted antigen treatment in the piglet model may helpful to guide the prevention and control of viral diarrhoea in children.

Taken together, our results demonstrate that RA provides a great opportunity for novel vaccine designs. RA interacts with DCs to guide lymphocyte migration to the intestinal tract, which enhances both mucosal and systemic immunity and is thus beneficial in preventing TGEV transmission between piglets and reducing the risk of diarrhoea.

## Additional Information

**How to cite this article**: Chen, X. *et al.* Retinoic acid facilitates inactivated transmissible gastroenteritis virus induce CD8^+^ T cells migrating to the porcine gut. *Sci. Rep.* **6**, 24152; doi: 10.1038/srep24152 (2016).

## Figures and Tables

**Table 1:** Immunization used for this study.

Groups	Immunization
Control	subcutanoues route with corn oil
RA s.c.	subcutanoues route with RA
TGEV s.c.	subcutanoues route with WI-TGEV
RA-TGEV s.c.	subcutanoues route with RA and WI-TGEV
TGEV oral	orally immunized with WI-TGEV
